# Association of sleep quality with duty hours, mental health, and medical errors among Japanese postgraduate residents: a cross-sectional study

**DOI:** 10.1038/s41598-024-51353-8

**Published:** 2024-01-17

**Authors:** Kazuya Nagasaki, Hiroyuki Kobayashi, Yuji Nishizaki, Masaru Kurihara, Takashi Watari, Taro Shimizu, Yu Yamamoto, Kiyoshi Shikino, Sho Fukui, Sho Nishiguchi, Kohta Katayama, Yasuharu Tokuda

**Affiliations:** 1 Department of Internal Medicine, Mito Kyodo General Hospital, University of Tsukuba, 3-2-7, Miyamachi, Mito, Ibaraki 310-0015 Japan; 2 Division of Medical Education, Juntendo University School of Medicine, Tokyo, Japan; 3 Department of Patient Safety, Nagoya University Hospital, Aichi, Japan; 4 General Medicine Center, Shimane University Hospital, Shimane, Japan; 5 Department of Diagnostic and Generalist Medicine, Dokkyo Medical University Hospital, Tochigi, Japan; 6 Division of General Medicine, Center for Community Medicine, Jichi Medical University, Tochigi, Japan; 7 Department of General Medicine, Chiba University Hospital, Chiba, Japan; 8 Department of Emergency and General Medicine, Kyorin University, Tokyo, Japan; 9 Department of General Internal Medicine, Shonan Kamakura General Hospital, Kamakura, Japan; 10 Department of General Internal Medicine, St. Marianna University School of Medicine, Kanagawa, Japan; 11 Department of Clinical Epidemiology, Graduate School of Medicine, Fukushima Medical University, Fukushima, Japan; 12 Muribushi Okinawa for Teaching Hospitals, Okinawa, Japan; 13 Tokyo Foundation for Policy Research, Tokyo, Japan

**Keywords:** Occupational health, Health policy

## Abstract

Long duty hours (DH) impair sleep and negatively affect residents’ health and medical safety. This cross-sectional study investigated the association among residents’ DH, sleep duration, insomnia, sleep impairment, depressive symptoms, and self-reported medical errors among 5579 residents in Japan who completed the General Medicine In-Training Examination (2021) and participated in the training-environment survey. Weekly DH was classified under seven categories. Sleep duration and insomnia symptoms, from the Athens Insomnia Scale, were analysed to determine sleep impairment; depressive symptoms and medical errors were self-reported. Among 5095 residents, 15.5% slept < 5 h/day, and 26.7% had insomnia. In multivariable analysis, compared with ≥ 60 and < 70, DH ≥ 90 h/week associated with shorter sleep duration and worsen insomnia symptoms. Shorter durations of sleep and more intense symptoms of insomnia were associated with increased depressive symptoms. Medical errors increased only among residents with insomnia, but were not associated with sleep duration. DH > 90 h/week could lead to shorter sleep duration, worsen insomnia symptoms, and negatively impact well-being and medical safety. There was no significant association between sleep duration and medical errors; however, insomnia conferred an increased risk of medical errors. Limiting DH for residents to avoid excessive workload can help improve resident sleep, enhance resident well-being, and potentially reduce insomnia-associated medical errors.

## Introduction

Long working hours can adversely affect health and increase the risk of mental disorders, suicide, sleep impairment, and cardiovascular disease^1–3^. Japanese resident-physicians often work long hours, and resident doctors in their 20s work, on average, 76.1 h/week^4^. A new legislation to limit overtime work to 1860 h per year (i.e., weekly duty hour [DH] limit: ~ 80 h/week) will apply from 2024 in Japan and was enacted based on the health–education balance and work regulations in other countries^5^. Nonetheless, the DH limit is significantly longer than those for general physicians (60 h/week) and general workers (48 h/week) and thus instil concern about the potential negative effects of work hours on health and lifestyle. A cross-sectional study on mental health among approximately one-third of the Japanese residents found that working > 90 h/week increased depression, burnout, and stress^6^. Longer DHs are associated with greater increases in depressive symptoms^7,8^. Decreasing DH or setting appropriate thresholds may improve mental health. Despite the reported benefits of DH restrictions on sleep-related impairment, the extent to which limiting weekly DH decreases resident sleep deprivation and improves well-being and patient safety remains unclear.

Few studies have investigated the association of weekly DH with sleep duration; however, most have consistently demonstrated that longer DH correlated with shorter sleep duration. A study involving the general workforce made a comparison between those working > 55 h/week and those working 35–40 h/week, revealing that the former group had a 1.98 times higher likelihood of sleeping < 7 h^9^. In 2011, a study of 1241 Japanese residents reported that sleep duration decreased as weekly DHs increased^7^. The study further indicated that almost all residents got < 7 h of sleep, and those working > 80 h/week often slept < 6 h, with an average of 5.67 h. Another study found a strong negative correlation between resident sleep duration and weekly DH^10^. In randomized controlled trials, shortened single-shift work or protected sleep time resulted in improved sleep duration and decreased sleepiness^11–13^. To our knowledge, no study has investigated the association between insomnia-related symptoms and weekly DHs in residents.

Inadequate sleep affects attention and working memory^14,15^, thereby eliciting concern about patient safety. A large body of evidence indicates that both acute and chronic sleep deprivation affect cognitive performance^14,16^. Among healthy adults, chronic sleep deprivation, with sleep duration < 6 h/day, caused cognitive impairment equivalent to that induced by 2 days of sleeplessness^14^. In a large-sample study of physicians, severe sleep-related impairment was associated with increased medical errors^17^. However, decreasing only the shift-work duration produced conflicting results of increasing/decreasing medical errors^18,19^.

Despite the posited association between sleep impairment and poor mental health, only fewer large-scale studies have investigated this relationship. Resident burnout, which overlaps with depression, can impair academic performance and reduce the quality of medical care^20,21^. Trockel et al.^17^ found a strong correlation between sleep-related impairment and burnout in a large sample of physicians. Other studies revealed a link between sleep deprivation and burnout^22,23^. However, a large prospective observational study found no effect of differences in DH or daily sleep duration on resident burnout^24^.

Japan’s current focus is on weekly DH limits to promote the health and sleep of residents. However, previous studies on the impact of DH on resident sleep have mainly examined interventions that improve per-shift work rather than weekly DH^25^ and were mostly conducted in the United States after the introduction of the weekly “80-h rule” in 2004^26^; therefore, those studies did not provide sufficient evidence of the optimal duration of weekly DH for sufficient sleep in Japan. This large-sample study examined the association between resident weekly DH and sleep-related variables, sleep duration, and insomnia symptoms among residents in Japan and assessed the impact of sleep-related impairments on residents’ depressive symptoms and self-reported medical errors.

## Methods

### Study setting

This national, cross-sectional study enrolled postgraduate residents who completed the General Medicine In-training Examination (GM-ITE) in Japan between January 17 and January 30, 2022 and subsequently participated in a training environment survey.

The research consent form, shared with the survey, ascertained voluntary participation and averred anonymized responses. All participants read and signed the informed consent document before the survey. The study was approved by the Ethical Review Committee of JAMEP (No. 22-1-2) and was conducted in accordance with the Strengthening the Reporting of Observational Studies in Epidemiology (STROBE) reporting guidelines. Additionally, this study was conducted in accordance with the Declaration of Helsinki.

### Participants

The participants were postgraduate year (PGY)-1 and PGY-2 residents who completed the GM-ITE and voluntarily participated. Respondents without complete data on DH and sleep-related variables were excluded from the analysis.

After medical school, Japanese residents undergo 2-year residency training to acquire general medical skills at community and university hospital training sites based on a preferential hospital-matching system for mandatory (internal medicine, emergency medicine, surgery, paediatrics, obstetrics and gynaecology, psychiatry, and community medicine) and elective rotations^23,27^. During their residency training, residents are required to perform multiple emergency duty (ED) shifts each month, in addition to their regular departmental rotation training.

### Measurements

#### Resident characteristics

Participant characteristics, including age, sex, PGY, hospital type (community or university hospital), average number of assigned inpatients (i.e., the number of inpatients a resident is responsible for at one time), number of ED duties per month, and self-study time (SST) per day, were obtained in the survey. The translated survey questions are presented in the supplementary material (Appendix 1). Resident participants completed an electronic survey immediately following the GM-ITE.

#### Sleep duration and insomnia symptoms

The average daily sleep duration in the previous month was classified as: ≤ 4, 5, 6, 7, 8, and ≥ 9 h. Insomnia-related symptoms were assessed with the Athens Insomnia Scale (AIS; Japanese version)^28^, which comprises eight questions on insomnia symptoms experienced in the previous month (each scored 0–3 points and total score of 24 points). A score ≥ 6 was a positive indicator of insomnia, and the severity of insomnia symptoms was graded by the AIS score as absence (0–5 points), mild (6–9 points), moderate (10–15 points), and severe (16–24 points)^29^.

#### Weekly DH

Residents were asked about their weekly DH during the entire training period including hours worked on weekdays, weekends, and night emergency department (ED) duties. They reported their DHs using a 10-category scale, which was then categorized into seven groups: 1 (C1, < 50 h), 2 (C2, ≥ 50 and < 60 h), 3 (C3, ≥ 60 and < 70 h), 4 (C4, ≥ 70 and < 80 h), 5 (C5, ≥ 80 and < 90 h), 6 (C6, ≥ 90 and < 100 h), and 7 (C7, ≥ 100 h).

#### Depressive symptoms

Depressive symptoms were assessed with the 2-item Patient Health Questionnaire (PHQ-2; Japanese version)^30^, which ascertains loss of interest or pleasure and depressive mood over the past 2 weeks. Positive depression screening was indicated by a “yes” response to either of the two questions. The PHQ has adequate diagnostic capability to identify clinical depression (sensitivity 76%; specificity 87%)^31^.

#### Medical errors

Residents were asked to report the number of their medical errors (which occurred 0, 1, 2, 3, 4, or ≥ 5 times) during the past year that caused minor or severe disabilities or disadvantages for patients.

### Statistical analysis

The characteristics of residents and their work environments were summarized by the weekly DH category, with category-specific data on sleep duration and insomnia symptoms. Associations between resident sleep and DH, mental health, and medical errors were analysed using multivariable analysis wherein Category 3 (≥ 60 and < 70 h) was the reference group, because 60 h/week is the DH limit for general physicians^5^. These analyses are repeated, categorized by PGY.

#### Weekly DH and sleep-related variables

The association between weekly DH and sleep duration and between weekly DH and insomnia symptoms were analysed in a proportional odds regression model. To account for variability, the OR and 95% CI were estimated using generalized estimating equations (GEE) with the working independent correlation within hospitals. The analysis was adjusted for age, sex, PGY, hospital type, ED duties, assigned inpatients, SST, and depressive symptoms.

#### Resident sleep and depressive symptoms

The association between sleep-related variables (sleep duration and insomnia symptoms) and depressive symptoms was analysed using modified Poisson regression models with GEEs to account for hospital variability after adjustment for age, sex, PGY, hospital type, weekly DH, ED duties, assigned patients, and SST.

#### Resident sleep and medical errors

The association between sleep-related variables (sleep duration and insomnia symptoms) and medical errors (minor or severe) was analysed using proportional odds regression models with GEE to account for hospital variability after adjusting for age, sex, PGY, hospital type, weekly DH, ED duty, assigned patients, SST, and depressive symptoms.

In addition, structural equation models were constructed to test the direct and indirect associations between weekly DH, sleep-related variables, depressive symptoms, and severe medical errors, employing standardized estimates of effect and standard goodness-of-fit measures. These analyses were conducted using STATA version 15 (STATA Corporation, College Station, TX, USA).

## Results

### Basic characteristics

A total of 7681 residents completed the GM-ITE, and 5579 agreed to participate in this study. After excluding respondents with incomplete data, the final sample comprised 5095 residents.

The basic characteristics of the participants are detailed in Table 1. Of the participants, 31.8% were women, 49.5% were in PGY-2, and 18.3% were from university hospitals. The average age was 26 years (29.3%), and 11.3% were ≥ 30 years. Residents were most frequently assigned to night ED duties 3–5 times per month (71.4%), had 5–9 inpatients (55.1%), and spent 0–30 min/day (41.0%) in self-study.

**Table 1:** Characteristics of the participants categorized by weekly duty hours.

Characteristics	Total	Weekly duty hours*						
		C1	C2	C3	C4	C5	C6	C7
	N = 5095	N = 754	N = 1246	N = 1177	N = 689	N = 770	N = 249	N = 210
Age (%)								
24	3.3	2.4	2.7	4.0	5.1	2.3	2.8	4.3
25	20.4	17.5	20.0	23.9	21.4	19.4	19.7	14.8
26	29.4	25.2	30.6	29.1	29.5	32.1	29.3	28.1
27	20.9	21.6	20.4	20.5	21.4	20.4	23.7	20.5
28	9.5	11.0	8.8	8.9	8.7	10.9	9.2	10.0
29	5.1	6.1	5.5	4.2	4.9	4.3	4.4	8.6
30 or more	11.4	16.2	12.0	9.4	9.0	10.6	10.8	13.8
PGY-2 (%)^†^	49.5	51.1	49.2	46.0	50.9	48.7	55.0	57.6
Male sex (%)	68.2	64.6	69.8	70.4	66.5	64.4	67.9	78.1
Community hospital (%)^‡^	81.7	75.2	82.0	84.0	84.5	80.1	84.3	84.3
ED duty per month (%)								
None	3.5	9.6	4.3	1.8	2.0	1.9	0.4	1.4
1–2	15.5	28.2	17.7	13.0	10.3	12.7	8.1	6.7
3–5	71.6	58.1	72.9	76.8	76.9	72.7	71.8	61.0
6 or more	8.8	2.8	4.4	8.1	10.5	12.5	19.0	29.5
Unknown	0.6	1.3	0.6	0.3	0.3	0.1	0.8	1.4
Assigned inpatient (%)								
0–4	30.3	46.2	35.7	25.4	24.0	25.3	21.7	17.6
5–9	55.2	46.1	54.5	59.7	58.2	57.8	53.0	50.5
10–14	8.9	4.0	6.4	9.1	11.2	11.5	14.1	17.1
15 or more	2.7	0.9	0.9	2.6	3.2	3.4	7.6	11.0
Unknown	2.9	2.8	2.5	3.2	3.3	2.0	3.6	3.8
Self-study time per day (%)								
None	3.5	3.8	3.2	3.8	2.8	3.4	2.0	8.1
0–30 min	41.1	50.5	43.5	38.6	37.9	37.1	39.5	35.2
31–60 min	39.3	34.6	41.0	39.4	40.6	42.3	38.3	31.9
61–90 min	12.4	9.4	9.7	15.2	14.3	12.6	15.3	14.3
91 min or more	3.5	1.6	2.6	3.1	4.4	4.7	4.8	10.5

*The categories of weekly duty hours were as follows: Category 1 (C1, < 50 h), Category 2 (C2, ≥ 50 and < 60 h), Category 3 (C3, ≥ 60 and < 70 h), Category 4 (C4, ≥ 70 and < 80 h), Category 5 (C5, ≥ 80 and < 90 h), Category 6 (C6, ≥ 90 and < 100 h), and Category 7 (C7, ≥ 100 h).

^†^Postgraduate residents are either Postgraduate year (PGY)-1 or PGY-2.

^‡^Training hospitals are divided into community and university hospitals.

### Sleep duration and insomnia symptoms

**Figure 1:**
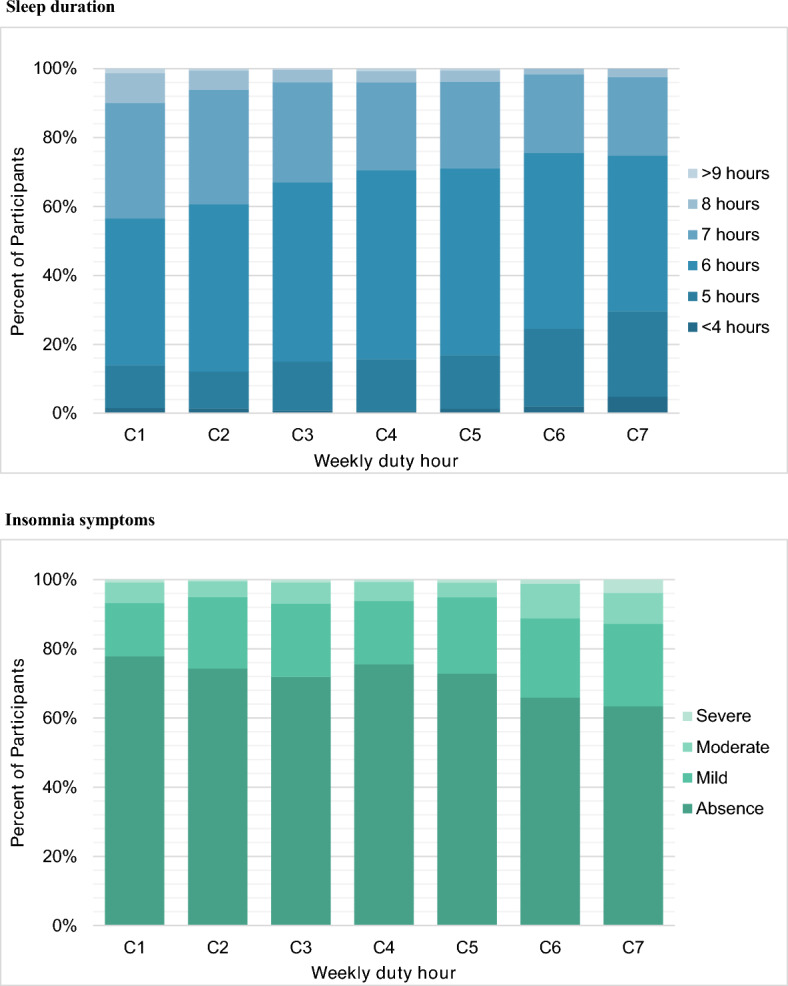
Sleep-related variables categorized by weekly duty hours. The categories of weekly duty hours were as follows: Category 1 (C1, < 50 h), Category 2 (C2, ≥ 50 and < 60 h), Category 3 (C3, ≥ 60 and < 70 h), Category 4 (C4, ≥ 70 and < 80 h), Category 5 (C5, ≥ 80 and < 90 h), Category 6 (C6, ≥ 90 and < 100 h), and Category 7 (C7, ≥ 100 h). *On the Athens Insomnia Scale, insomnia severity is classified as follows: 0–5 points, “absence”; 6–9 points, “mild”; 10–15 points, “moderate”; and 16–24 points, “severe”.

Figure 1 presents DH-category-specific data on resident sleep duration and insomnia symptoms. Approximately half of the residents (50.2%) reported sleeping 6 h/day, whereas 15.5% slept < 5 h/day. The group of residents with DH ≥ 80 h/week (C6, C7, and C8) comprised a higher proportion (20.6%) of participants who slept < 5 h/day, as compared to the proportion (12.7%) in the group of residents who worked < 60 h/week (C1 and C2).

The mean AIS score was 3.96 ± 3.7; 26.7% of residents had insomnia symptoms. Among them 20.2%, 5.6%, and 0.9% had mild, moderate, and severe symptoms, respectively. A significant proportion of residents who worked ≥ 80 h/week (30.2%) experienced moderate (6.2%) or severe (1.5%) insomnia symptoms.

### Weekly DH and sleep-related variables

Table 2 presents the OR of DH in the univariable and multivariable proportional odds models for sleep duration and insomnia symptoms. Multivariable analysis revealed that residents who worked < 60 h/week (Categories 1 and 2) had longer sleep duration than residents who worked ≥ 60 and < 70 h/week (Category 3; reference group). Conversely, residents who worked ≥ 80 h/week (Categories 5, 6, and 7) had a shorter sleep duration than residents in the reference group. The severity of insomnia symptoms was lower in the group that worked < 50 h/week (Category 1) and higher in the group that worked ≥ 90 and < 100 h/week (Category 6), as compared to the reference group.

**Table 2:** Association between weekly duty hours and resident sleep-related variables analysed using proportional odds regression models.

Duty hour category	N	Unadjusted		Adjusted*	
		Odds ratio (95% CI)	p-value	Odds ratio (95% CI)	p-value
Sleep duration					
C1: < 50 h	754	0.73 (0.59–0.91)	0.004	0.71 (0.59–0.91)	0.006
C2: ≥ 50 to < 60 h	1246	0.86 (0.72–1.03)	0.11	0.89 (0.73–1.08)	0.24
C3: ≥ 60 to < 70 h	1177	Reference			
C4: ≥ 70 to < 80 h	689	0.83 (0.67–1.03)	0.10	0.81 (0.64–1.02)	0.07
C5: ≥ 80 to < 90 h	770	0.93 (0.76–1.14)	0.48	0.91 (0.73–1.14)	0.41
C6: ≥ 90 to < 100 h	249	1.38 (1.03–1.84)	0.03	1.44 (1.06–1.97)	0.02
C7: ≥ 100 h	210	1.58 (1.16–2.15)	0.004	1.30 (0.93–1.82)	0.13
Insomnia symptoms					
C1: < 50 h	754	1.51 (1.26–1.80)	< 0.001	1.58 (1.32–1.90)	< 0.001
C2: ≥ 50 to < 60 h	1246	1.30 (1.12–1.51)	0.001	1.31 (1.13–1.53)	< 0.001
C3: ≥ 60 to < 70 h	1177	Reference			
C4: ≥ 70 to < 80 h	689	0.89 (0.75–1.07)	0.21	0.91 (0.76–1.09)	0.31
C5: ≥ 80 to < 90 h	770	0.86 (0.72–1.02)	0.09	0.90 (0.75–1.07)	0.22
C6: ≥ 90 to < 100 h	249	0.60 (0.47–0.78)	< 0.001	0.63 (0.49–0.82)	0.00
C7: ≥ 100 h	210	0.53 (0.40–0.71)	< 0.001	0.58 (0.44–0.78)	< 0.001

The association between DHs and sleep durations, as well as between DHs and insomnia symptoms were analysed using proportional odds regression models with generalized estimating equations to account for hospital variability. The reference group for the analysis was duty hour category 3 (≥ 60 to 70 h/week). Sleep duration was categorized into six categories per day: ≤ 4, 5, 6, 7, 8, and ≥ 9 h. Insomnia symptoms was classified by scores on the Athens Insomnia Scale, with 0–5 points as “absence”, 6–9 points as “mild”, 10–15 points as “moderate”, and 16–24 points as “severe”.

*CI* confidence interval.

*Both models are adjusted for age, sex, postgraduate year, hospital type, emergency department duty, assigned inpatient, self-study time, and depressive symptoms.

The detailed results of the multivariable proportional odds models are presented in Supplementary Table 1. The analysis revealed that older, male, and PGY-1 residents were more likely to experience shorter sleep durations and insomnia symptoms. University hospital residents showed a higher likelihood of experiencing insomnia symptoms than community hospital residents. The number of emergency duty shifts did not significantly affect resident sleep; however, having more inpatients under their care reduced sleep duration. Increased self-study was associated with shorter sleep duration but fewer insomnia symptoms. Finally, depressive symptoms were linked to shorter sleep duration and a significantly higher likelihood of insomnia symptoms.

### Resident sleep and depressive symptoms

Table 3 presents the prevalence ratio (PR) of sleep-related variables in the univariable and multivariable modified Poisson regression models for the presence of depressive symptoms. The prevalence of depressive symptoms by category of sleep-related variables is shown in Supplementary Table 2. The prevalence of depressive symptoms was 24.4%. The results of the multivariable analysis indicate that individuals who slept < 4 or 5 h/day had a higher prevalence of depressive symptoms than those who slept 7 h/day (PR [95% CI]: 2.12 [1.66–2.72] and 1.56 [1.37–1.78], respectively). Compared to those without insomnia symptoms, residents who experienced insomnia symptoms were 3–5 times more likely to have depressive symptoms.

**Table 3:** Association between resident sleep and depressive symptoms analysed using modified Poisson regression models.

Duty hour category	N	Unadjusted		Adjusted*	
		Prevalence ratio (95% CI)	p-value	Prevalence ratio (95% CI)	p-value
Sleep duration					
< 4 h	67	2.57 (2.01–3.28)	< 0.001	2.12 (1.66–2.72)	< 0.001
5 h	726	1.68 (1.47–1.92)	< 0.001	1.56 (1.37–1.78)	< 0.001
6 h	2556	1.08 (0.96–1.22)	0.19	1.05 (0.94–1.18)	0.39
7 h	1483	Reference			
8 h	232	1.13 (0.89–1.45)	0.31	1.11 (0.87–1.41)	0.42
> 9 h	31	1.70 (1.03–2.78)	0.04	1.64 (0.97–2.76)	0.06
Insomnia symptoms^†^					
Absence	3737	Reference			
Mild	1027	3.20 (2.87–3.56)	< 0.001	3.10 (2.77–3.47)	< 0.001
Moderate to severe	331	5.12 (4.60–5.70)	< 0.001	4.86 (4.35–5.42)	< 0.001

The association between sleep-related variables (sleep duration and insomnia symptoms) and depressive symptoms were analysed using modified Poisson regression models with generalized estimating equations to account for hospital variability. Depressive symptoms were measured dichotomously with the Patient Health Questionnaire (PHQ)-2.

*CI* confidence interval.

*Adjusted for age, sex, postgraduate year, hospital type, weekly duty hour, emergency department duty, assigned inpatient, and self-study time.

^†^Insomnia symptoms was classified by scores on the Athens Insomnia Scale, with 0–5 points as “absence”, 6–9 points as “mild”, 10–15 points as “moderate”, and 16–24 points as “severe”. For the analysis, the “moderate” and “severe” categories were combined due to the small number of participants in the “severe” category (N = 45).

### Resident sleep and medical errors

Table 4 presents the OR of sleep-related variables in the univariable and multivariable proportional odds models for both minor and severe medical errors. The number of medical errors per year per category of sleep–related variables is shown in Supplementary Table 3. Overall, 45.9% and 18.7% of residents had experienced at least one minor and one severe error, respectively, in the past year. Multivariate analysis revealed no clear association between medical errors and sleep duration, although the presence of insomnia symptoms was linked to an increase in medical errors. Compared to residents without insomnia symptoms, those with mild and moderate to severe insomnia symptoms had higher rates of minor medical errors (OR [95% CI]: 1.21 [1.05–1.40] and 1.42 [1.13–1.81], respectively), whereas those with moderate to severe insomnia symptoms had higher rates of severe errors (1.92 [1.45–2.56]).

**Table 4:** Association between resident sleep-related variables and medical errors analysed using proportional odds regression models.

Minor error					
Duty hour category	N	Unadjusted		Adjusted*	
		Odds ratio (95% CI)	p-value	Odds ratio (95% CI)	p-value
Sleep duration					
< 4 h	67	1.09 (0.67–1.77)	0.72	1.00 (0.61–1.65)	1.00
5 h	726	0.93 (0.78–1.11)	0.43	0.93 (0.77–1.11)	0.39
6 h	2556	0.95 (0.84–1.08)	0.43	0.95 (0.84–1.08)	0.42
7 h	1483	Reference			
8 h	232	1.07 (0.81–1.41)	0.63	1.13 (0.86–1.49)	0.38
> 9 h	31	0.71 (0.33–1.51)	0.37	0.73 (0.34–1.57)	0.43
Insomnia symptoms^†^					
Absence	3737	Reference			
Mild	1027	1.28 (1.12–1.47)	< 0.001	1.21 (1.05–1.40)	0.01
Moderate to severe	331	1.57 (1.26–1.96)	< 0.001	1.42 (1.13–1.81)	0.004

Severe error					
Duty hour category	N	Unadjusted		Adjusted*	
		Odds ratio (95% CI)	p-value	Odds ratio (95% CI)	p-value
Sleep duration					
< 4 h	67	1.64 (0.92–2.93)	0.09	1.50 (0.83–2.70)	0.19
5 h	726	0.86 (0.68–1.09)	0.22	0.82 (0.64–1.05)	0.10
6 h	2556	0.90 (0.76–1.07)	0.22	0.90 (0.76–1.06)	0.21
7 h	1483	Reference			
8 h	232	1.12 (0.79–1.59)	0.53	1.14 (0.80–1.62)	0.48
> 9 h	31	2.69 (1.25–5.77)	0.01	2.70 (1.25–5.80)	0.01
Insomnia symptoms^†^					
Absence	3737	Reference			
Mild	1027	1.27 (1.06–1.52)	0.01	1.17 (0.97–1.42)	0.10
Moderate to severe	331	2.40 (1.82–3.05)	< 0.001	1.92 (1.45–2.56)	< 0.001

*CI* confidence interval.

The association between sleep-related variables (sleep duration and insomnia symptoms) and medical errors were analysed using modified Poisson regression models with generalized estimating equations to account for hospital variability. The number of medical errors that resulted in minor or severe disability or disadvantage to patients experienced in the past year were reported based on the following numbers: 0, 1, 2, 3, 4, or 5 or more times.

*Adjusted for age, sex, postgraduate year, hospital type, weekly duty hours, emergency department duty, assigned inpatient, self-study time, and depressive symptoms.

^†^Insomnia symptoms was classified by scores on the Athens Insomnia Scale, with 0–5 points as “absence”, 6–9 points as “mild”, 10–15 points as “moderate”, and 16–24 points as “severe”. For the analysis, the “moderate” and “severe” categories were combined due to the small number of participants in the “severe” category (N = 45).

### Analyses categorized by postgraduate year (PGY)

The results of multivariable analyses assessing the associations between resident sleep and DH, mental health, and medical errors, categorized by PGY, were presented in Supplementary Tables 3, 4, and5. Although no significant differences were observed in the association between DH and sleep duration across different PGYs, insomnia symptoms were positively associated with DH exclusively in PGY-1 residents, unlike in PGY-2 residents. The associations between sleep-related variables and depressive symptoms, as well as between sleep-related variables and medical errors, were similar for both PGY-1 and PGY-2 residents.

### Path analysis

**Figure 2:**
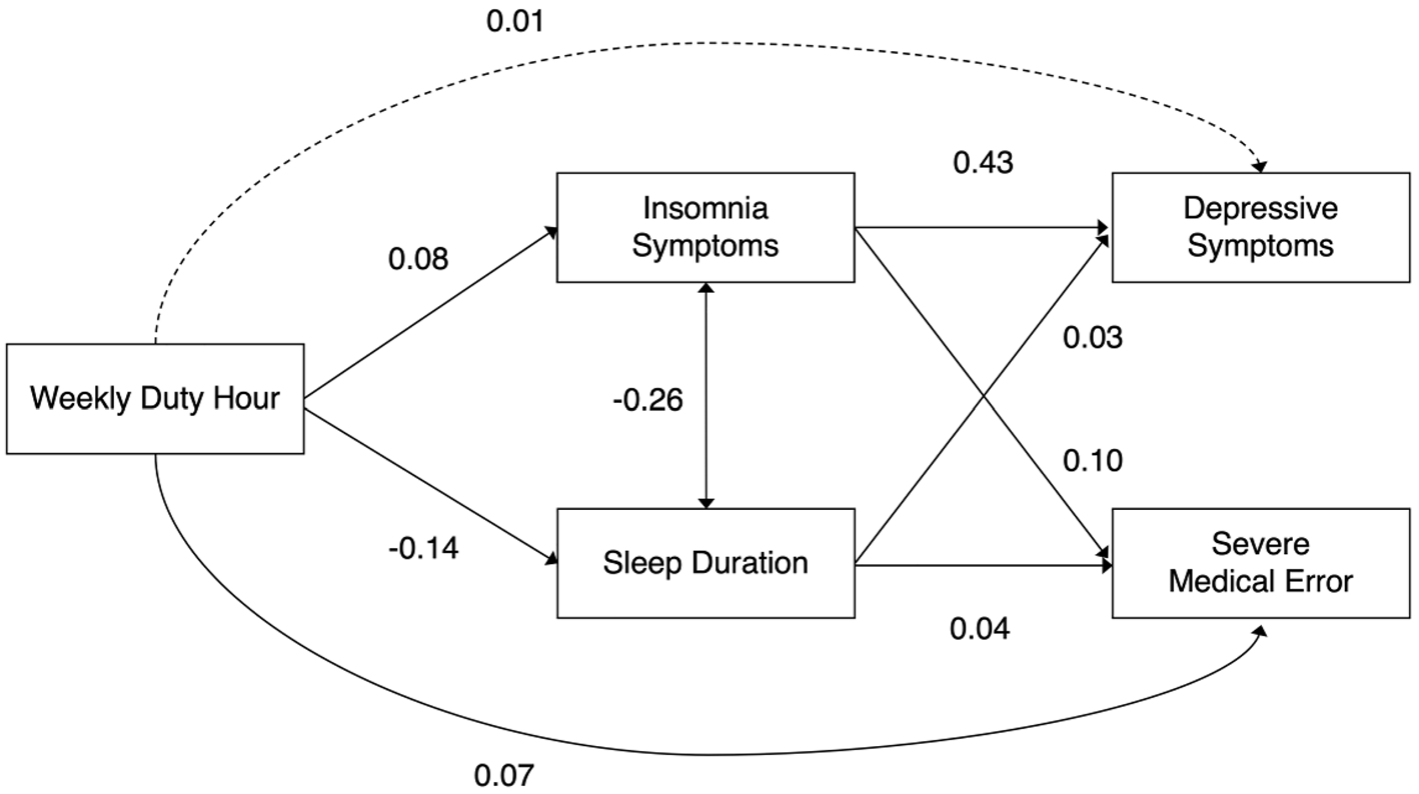
Results of structural equation modeling among the association between weekly duty hours, sleep-related variables, depressive symptoms, and severe medical error. Insomnia symptoms and sleep duration are considered as covariates. The values shown are standardized profit regression coefficients. Solid lines indicate statistical significance (p < 0.05), while dashed lines represent relationships that are not statistically significant. Our model was well fitted to our data (root mean square error of approximation [RMSEA]: 0.048; comparative fit index [CFI]: 0.99; Tucker-Lewis index [TLI]: 0.93).

Figure 2 summarizes the results of the path analysis conducted using SEM to examine the associations among weekly duty hours, sleep-related variables, depressive symptoms, and severe medical errors. Depressive symptoms were directly influenced by insomnia symptoms (β [95% CI]: 0.43 [0.41–0.46]) and sleep duration (0.03 [0.001–0.05]), with insomnia symptoms having a greater effect. Similarly, medical errors were directly affected by insomnia symptoms (0.10 [0.07–0.13]) and sleep duration (0.05 [0.02–0.07]), with insomnia symptoms having a larger effect. Weekly duty hours had an indirect effect on these effects (to insomnia symptoms: 0.08 [0.05–0.11], to sleep duration: − 0.14 [− 0.17 to − 0.11]). In addition to this indirect effect, we also modeled a direct effect of duty hours on depressive symptoms and medical errors. However, the direct effect on depressive symptoms was not statistically significant (0.01 [− 0.01 to 0.04]), while the effect on medical errors was significant (0.07 [0.04–0.09]). Our model demonstrated a good fit to our data, as indicated by the root mean square error of approximation (RMSEA) of 0.048, the comparative fit index (CFI) of 0.99, and the Tucker–Lewis index (TLI) of 0.93. The result of another path analysis, which replaced severe medical error with minor medical error, is presented in Supplementary Fig. 1. The results of the path analysis with minor errors were identical to those with severe medical errors.

## Discussion

This cross-sectional study, conducted in Japan, included 5095 PGY-1 and PGY-2 residents and investigated the association between resident DH, sleep, and sleep-related outcomes. The results showed that 15.5% of the residents had chronic sleep deprivation (sleep duration < 5 h/day), and 26.7% had insomnia symptoms. Multivariable analysis indicated that DH > 90 h/week were associated with shorter sleep duration and increased severity of insomnia symptoms. However, sleep duration was prolonged when DH was < 60 h/week, and the severity of insomnia symptoms decreased when DH was < 50 h/week. Shorter sleep durations (< 4 or 5 h/night) and more severe insomnia symptoms were significantly associated with depressive symptoms, and insomnia symptoms were linked to a greater number of medical errors, regardless of severity. Surprisingly, sleep duration did not affect the number of errors.

This study revealed that > 90 h weekly DH was associated with reduced sleep duration and heightened insomnia symptoms among residents. This is an important discovery as Japan intends to restrict residents’ DH imminently in 2024, wherein the maximum weekly DH for residents is expected to be 80 h, which may improve sleep quality. Furthermore, restricting DH to ≤ 60 h/week, as for general physicians, may increase sleep duration and alleviate insomnia symptoms. However, the disparity between the 80-h and 60-h weekly limit is not substantial. Therefore, when determining the degree to which work hours should be reduced to enhance sleep quality, various perspectives should be considered, including resident education, well-being, and patient care quality.

In this study, various factors that affect residents’ sleep were examined. First, the study discovered that the number of additional ED duties in they did not affect resident sleep although the average number of inpatients assigned to a resident at one time did. This implies that chronic sleep deprivation resulting from a demanding daily workload may have a more significant effect on sleep than acute sleep deprivation resulting from long shifts. Second, several specific resident characteristics, including older age, PGY-1 level, male sex, and university hospital affiliation, were associated with poor sleep quality. Third, the study showed that the duration of self-study among residents was linked to shorter sleep duration; however, paradoxically, they also had fewer insomnia symptoms. This may be because residents without insomnia can study for extended periods.

In this study, short sleep duration and insomnia symptoms were clearly related to depressive symptoms. Approximately 20% of the patients with insomnia experience depression^32^. A recent large cohort study of US medical interns found that longer weekly DH were associated with a greater increase in depressive symptoms from baseline^8^. The rate of depression among resident physicians in Japan is comparable to healthcare professionals in ICUs during the COVID-19 pandemic, necessitating depression reduction measures^33^. Potentially, reducing the number of hours worked per week may improve sleep and well-being of residents.

Notably, the number of medical errors experienced by residents was associated with insomnia symptoms but not with sleep duration. A similar finding was reported in a 2019 study of general workers, where insomnia symptoms, but not sleep duration, were linked to cognitive errors in the workplace^34^. This contrasts with several previous reports that found an association between sleep duration and cognitive errors^35,36^. For instance, a prospective study involving 1215 depression-free interns who slept < 6 h/night reported a higher rate of medical errors^36^. The reason for this inconsistency might be attributed to variations in job responsibilities, work scope, and degrees of sleep deprivation. In this study, no significant difference was observed in the association between medical errors and sleep duration among PGY-1 and PGY-2 residents. However, such an association might exist among physicians who typically bear more responsibility in high-intensity clinical settings. Insomnia appears to be particularly linked to an increase in medical errors, underscoring the importance of interventions to improve sleep quality. Reducing the DH to < 90 h/week may mitigate the exacerbation of insomnia symptoms and reduce medical errors. However, our data suggest that reducing DHs beyond 90 h/week does not make much of a difference regarding the presence or absence of insomnia. Therefore, it may be beneficial to consider additional approaches to address insomnia beyond simply reducing DH.

This study recommends several ways to improve the working environments of residents in Japan. First, weekly DH should be limited to < 90 h/week because working more has been shown to worsen chronic sleep deprivation and insomnia. Specifically, our data suggest that interventions targeting insomnia symptoms might be particularly beneficial for PGY-1 residents. The upcoming implementation of a weekly DH limit of 80 h/week for residents by 2024 aligns with this recommendation. However, as residents often express a willingness to exceed this limit, program leaders should provide adequate explanation and monitoring^37^. Second, reducing daily workloads (e.g., assigned inpatients) may be more effective in improving sleep quality than reducing irregular duties (e.g., ED duty shifts). Though some hospitals focus on reducing weekly DH by decreasing ED duties, this intervention may not be sufficient to address sleep impairment. Third, to effectively reduce medical errors, it is necessary to implement a multifaceted intervention targeting insomnia symptoms rather than simply maintaining sleep duration. Clinical training hospital directors should identify residents at high risk for insomnia and monitor their symptoms closely. Moreover, screening at entry may be useful, as a previous report indicates that 5–10% of residents have insomnia symptoms at the start of training^38^.

This study had several limitations. First, all data on resident DH, sleep, medical safety, and depressive symptoms were self-reported and may have been subject to response bias. In particular, reports of medical errors may be influenced by social desirability bias. To mitigate potential bias, we highlighted in the electronic questionnaire that the survey responses would be anonymous and would not impact the examination results. Second, it was not possible to specify a causal relationship between each outcome and sleep status. Depressive symptoms can cause sleep disturbances, and prolonged sleep deprivation can lead to depressive symptoms, which makes it difficult to determine causality^32^. We interpreted the study results as an increase in medical errors based on the presence or absence of insomnia symptoms. However, data on medical errors and insomnia symptoms were collected at different times (within 1 year and 1 month, respectively). Therefore, to interpret these results, we must assume that insomnia symptoms are chronic. Future follow-up studies are warranted to monitor resident sleep and medical errors. Third, there is a possibility of selection bias. The sample of residents in this study was limited to 18.2% of those affiliated with university hospitals, although university-affiliated residents comprise approximately half of all residents in the country. This study indicated that university-affiliated residents had shorter sleep durations than community hospital residents, which may have affected the overall results. Fourth, the limited scope of the survey questions precluded a detailed understanding of the nature of the medical errors experienced by the residents. Consequently, there is insufficient data to assess how improvements in resident sleep might benefit patient care.

## Conclusions

Working > 90 h/week was associated with short sleep duration and greater insomnia symptoms among residents. Both short sleep duration and insomnia symptoms were associated with higher levels of depression among residents, whereas only insomnia symptoms were associated with increased medical errors. Thus, the 80-h workweek limit in Japan may potentially improve residents’ sleep, improve well-being, and promote medical safety. However, additional improvements could be achieved through sleep interventions that go beyond focusing solely on reducing work hours.

### Supplementary Information

Supplementary Information.

## Data Availability

The datasets generated during and/or analysed during the current study are not publicly available due to the nature of this research but are available from the corresponding author on reasonable request.
