# Supplementary material for: Association of sleep quality with duty hours, mental health, and medical errors among Japanese postgraduate residents: a cross-sectional study
Source: Sci Rep. 2024 Jan 17;14:1481. doi: 10.1038/s41598-024-51353-8 (PMC10794685; doi:10.1038/s41598-024-51353-8)
Supplement: Supplementary file 1 — Supplementary Information. [file 41598_2024_51353_MOESM1_ESM.docx]

**Supplementary material for “Association of Sleep Quality With Duty Hours, Mental Health, and Medical Errors Among Japanese Postgraduate Residents: A Cross-sectional Study”**

**This supplementary material comprises the following:**

**Appendix 1.** The survey inquiries featured in the GM-ITE questionnaire.

**Supplement Table 1.** Factors related to sleep-related variables (multivariable analysis).

**Supplementary Table 2**. Depressive symptoms categorized by sleep duration and insomnia symptoms

**Supplementary Table 3**. Medical errors categorized by sleep duration and insomnia symptoms

**Supplementary Table 4.** Association between weekly duty hours and resident sleep-related variables analysed using proportional odds regression models, categorized by postgraduate year

**Supplementary Table 5.** Association between resident sleep and depressive symptoms analysed using modified Poisson regression models, categorized by postgraduate year

**Supplementary Figure 1.** Results of structural equation modeling among the association between weekly duty hours, sleep-related variables, depressive symptoms, and minor medical error

**Appendix 1**. The survey inquiries featured in the GM-ITE questionnaire.

Please answer the following questions:

**Average number of assigned inpatients on regular basis**

(1) 0–4 (2) 5–9 (3) 10–14 (4) 15 or more (5) Unknown

**Average number of emergency department duties**

(1) 0 (2) 1–2 (3) 3–5 (4) 6 or more (5) Unknown

**Average study time per day (self-study at a desk)**

(1) 0–30 min (2) 31–60 min (3) 61–90 min (4) 91 min or more (5) None

**Average duty hours per week** (including standby hours during emergency duty)

(1) <45 hours (2) ≥45–<50 hours (3) ≥50–<55 hours (4) ≥55–<60 hours

(5) ≥60–<65 hours (6) ≥65–<70 hours (7) ≥70–<80 hours (8) ≥80–<90 hours

(9) ≥90–<100 hours (10) ≥100 hours

Note: Example of calculation

Weekday duty hours (Monday–Friday, 8:30–19:00, excluding 1-hour breaks): 47.5 hours

Weekday night emergency duty (once per week): 13.5 hours

Weekend duty hours + night emergency duty (once per week): 24 hours

= 85 hours

**Sleep condition**

During the past month, on average, how many hours of sleep did you get per day?

(1) <4 hours (2) 5 hours (3) 6 hours (4) 7 hours(5) 8 hours (6) 9 hours (7) ≥10 hours

For the following questions, please select the option that applies to experiences occurring at least three times a week in the past month.

Did it take you longer to fall asleep after you went to bed?

(1) No problem (2) Slightly delayed (3) Markedly delayed (4) Very delayed or did not sleep at all

Did you wake up in the middle of sleep during the night?

(1) No problem (2) Minor problem (3) Considerable problem (4) Serious problem or did not sleep at all

Did you experience waking up earlier than your desired wake-up time and have trouble falling back asleep afterward?

(1) Not earlier (2) Little earlier (3) Markedly earlier (4) Much earlier or did not sleep at all

Including night sleep and naps, did you get enough sleep?

(1) Sufficient (2) Slightly insufficient (3) Markedly insufficient (4) Very insufficient or did not sleep at all

How do you feel about the overall quality of your sleep?

(1) Satisfactory (2) Slightly unsatisfactory (3) Markedly unsatisfactory (4) Very unsatisfactory or could not sleep at all

How did you feel during the day?

(1) Normal (2) Slightly depressed (3) Markedly depressed (4) Very depressed

How were your physical and mental activities during the day?

(1) Normal (2) Slightly decreased (3) Markedly decreased (4) Very decreased

Did you feel sleepy during the day?

(1) None (2) Mild (3) Considerable (4) Intense

**Depression**

Have you been feeling down or depressed often during the past month?

1. Yes
2. No

Have you often felt uninterested or not very pleased with doing things during the past month?

1. Yes
2. No

**Scoring**

If either question is yes, the respondent is considered positive for depression screening.

**Medical error**

Please indicate the number of cases of severe disability or disadvantage to patients resulting from errors in your own medical practice within one year.

(1) 0 (2) 1 (3) 2 (4) 3 (5) 4 (6) 5 or more

Please indicate the number of cases of minor disability or disadvantage to patients resulting from errors in your own medical practice within one year.

(1) 0 (2) 1 (3) 2 (4) 3 (5) 4 (6) 5 or more

**Supplemental Table 1.** Factors related to sleep-related variables (multivariable analysis)

|  | **Sleep duration*** | | | **Insomnia symptoms†** | | |
| --- | --- | --- | --- | --- | --- | --- |
|  | **OR** | **95%CI** | | **OR** | **95%CI** | |
| Age |  |  |  |  |  |  |
| 24 | Reference |  |  | Reference |  |  |
| 25 | 1.01 | 0.74 | 1.38 | 1.04 | 0.70 | 1.55 |
| 26 | 0.99 | 0.73 | 1.35 | 0.99 | 0.67 | 1.48 |
| 27 | 0.91 | 0.66 | 1.25 | 1.10 | 0.73 | 1.67 |
| 28 | 0.79 | 0.56 | 1.12 | 0.98 | 0.62 | 1.53 |
| 29 | 0.74 | 0.51 | 1.08 | 1.59 | 0.99 | 2.55 |
| 30 or more | 0.58 | 0.41 | 0.81 | 1.73 | 1.13 | 2.64 |
| Men (vs. women) | 1.21 | 1.08 | 1.36 | 1.23 | 1.06 | 1.43 |
| PGY-2 (vs. PGY-1) | 1.18 | 1.05 | 1.33 | 0.85 | 0.74 | 0.99 |
| Community hospital (vs. university hospital) | 1.34 | 1.14 | 1.57 | 0.81 | 0.66 | 1.00 |
| ED duty per month |  |  |  |  |  |  |
| None | Reference |  |  | Reference |  |  |
| 1–2 | 1.03 | 0.75 | 1.41 | 0.79 | 0.53 | 1.17 |
| 3–5 | 1.10 | 0.81 | 1.49 | 0.90 | 0.62 | 1.32 |
| 6 or more | 0.91 | 0.64 | 1.30 | 1.11 | 0.71 | 1.72 |
| Unknown | 1.44 | 0.64 | 3.22 | 1.19 | 0.51 | 2.76 |
| Assigned inpatient |  |  |  |  |  |  |
| 0–4 | Reference |  |  | Reference |  |  |
| 5–9 | 0.96 | 0.85 | 1.08 | 1.03 | 0.88 | 1.21 |
| 10–14 | 0.84 | 0.68 | 1.04 | 1.18 | 0.90 | 1.53 |
| 15 or more | 0.60 | 0.42 | 0.86 | 1.10 | 0.73 | 1.66 |
| Unknown | 0.59 | 0.42 | 0.82 | 1.66 | 1.13 | 2.45 |
| Self-study time per day |  |  |  |  |  |  |
| None | Reference |  |  | Reference |  |  |
| 0–30 min | 0.80 | 0.59 | 1.08 | 0.75 | 0.53 | 1.06 |
| 31–60 min | 0.70 | 0.51 | 0.94 | 0.71 | 0.50 | 1.01 |
| 61–90 min | 0.69 | 0.50 | 0.96 | 0.67 | 0.46 | 0.99 |
| 91 min or more | 0.56 | 0.37 | 0.85 | 0.56 | 0.34 | 0.91 |
| Depressive symptoms (vs. no symptoms) | 0.70 | 0.62 | 0.80 | 6.81 | 5.90 | 7.85 |

Note: The association between DHs and sleep durations, as well as between DHs and insomnia symptoms were analysed using proportional odds regression models with generalized estimating equations to account for hospital variability. Both models are adjusted for age, sex, postgraduate year, hospital type, emergency department duty, assigned patient, self-study time, and depressive symptoms. The reference group for the analysis was duty hour category 3 (≥60 to <70 hours/week).

* Sleep duration was categorized into six categories per day: ≤4, 5, 6, 7, 8, and ≥9 hours.

†Insomnia symptoms was classified by scores on the Athens Insomnia Scale, with 0–5 points as “absence”, 6–9 points as “mild”, 10–15 points as “moderate”, and 16–24 points as “severe”.

Abbreviations: OR = odds ratio; CI = confidence interval.

**Supplemental Table 2.** Depressive symptoms categorized by sleep duration and insomnia symptoms

|  |  |  |
| --- | --- | --- |
| **Variables** | **N** | **Prevalence of having depressive symptoms (%)** |
| Sleep duration |  |  |
| < 4 hours | 67 | 53.7 |
| 5 hours | 726 | 35.1 |
| 6 hours | 2,556 | 22.6 |
| 7 hours | 1,483 | 20.9 |
| 8 hours | 232 | 23.7 |
| > 9 hours | 31 | 35.5 |
| Insomnia symptoms* |  |  |
| Absence | 3,737 | 14.3 |
| Mild | 1,027 | 45.7 |
| Moderate | 331 | 73.1 |

Note: Depressive symptoms were measured dichotomously with the Patient Health Questionnaire (PHQ)-2.

*Insomnia symptoms was classified by scores on the Athens Insomnia Scale, with 0–5 points as “absence”, 6–9 points as “mild”, 10–15 points as “moderate”, and 16–24 points as “severe”. For the analysis, the “moderate” and “severe” categories were combined due to the small number of participants in the “severe” category (N = 45).

**Supplemental Table 3.** Medical errors categorized by sleep duration and insomnia symptoms

|  |  | **Minor medical error (%)** | | | | | |
| --- | --- | --- | --- | --- | --- | --- | --- |
| **Variables** | **N** | **0** | **1** | **2** | **3** | **4** | **> 5** |
| Sleep duration |  |  |  |  |  |  |  |
| < 4 hours | 67 | 1.2% | 1.4% | 0.4% | 2.5% | 9.5% | 2.0% |
| 5 hours | 726 | 14.6% | 13.6% | 14.4% | 12.1% | 19.0% | 15.6% |
| 6 hours | 2,556 | 50.2% | 51.0% | 49.6% | 53.3% | 42.9% | 42.2% |
| 7 hours | 1,483 | 28.7% | 29.0% | 30.0% | 27.6% | 23.8% | 34.4% |
| 8 hours | 232 | 4.5% | 4.4% | 5.2% | 4.0% | 0.0% | 5.3% |
| >9 hours | 31 | 0.7% | 0.4% | 0.4% | 0.5% | 4.8% | 0.4% |
| Insomnia symptoms* |  |  |  |  |  |  |  |
| Absence | 3,737 | 75.4% | 73.6% | 70.6% | 68.8% | 57.1% | 60.2% |
| Mild | 1,027 | 18.9% | 20.1% | 22.5% | 20.6% | 19.0% | 27.9% |
| Moderate to severe | 331 | 5.6% | 6.3% | 6.9% | 10.6% | 23.8% | 11.9% |

|  |  | **Severe medical error (%)** | | | | | |
| --- | --- | --- | --- | --- | --- | --- | --- |
| **Variables** | **N** | **0** | **1** | **2** | **3** | **4** | **> 5** |
| Sleep duration |  |  |  |  |  |  |  |
| < 4 hours | 67 | 1.2% | 1.1% | 2.0% | 4.3% | 13.3% | 6.2% |
| 5 hours | 726 | 14.5% | 12.6% | 13.8% | 18.8% | 6.7% | 12.5% |
| 6 hours | 2,556 | 50.6% | 50.1% | 45.3% | 42.0% | 40.0% | 50.0% |
| 7 hours | 1,483 | 28.8% | 30.5% | 31.0% | 30.4% | 33.3% | 21.9% |
| 8 hours | 232 | 4.4% | 5.1% | 5.4% | 2.9% | 0.0% | 9.4% |
| >9 hours | 31 | 0.5% | 0.6% | 2.5% | 1.4% | 6.7% | 0.0% |
| Insomnia symptoms* |  |  |  |  |  |  |  |
| Absence | 3,737 | 74.7% | 70.9% | 64.5% | 50.7% | 46.7% | 62.5% |
| Mild | 1,027 | 19.7% | 21.9% | 21.7% | 24.6% | 20.0% | 21.9% |
| Moderate to severe | 331 | 5.6% | 7.2% | 13.8% | 24.6% | 33.3% | 15.6% |

Note: The number of medical errors that resulted in minor or severe disability or disadvantage to patients experienced in the past year were reported based on the following numbers: 0, 1, 2, 3, 4, or 5 or more times.

*Insomnia symptoms was classified by scores on the Athens Insomnia Scale, with 0–5 points as “absence”, 6–9 points as “mild”, 10–15 points as “moderate”, and 16–24 points as “severe”. For the analysis, the “moderate” and “severe” categories were combined due to the small number of participants in the “severe” category (N = 45).

**Supplementary Table 4.** Association between weekly duty hours and resident sleep-related variables analysed using proportional odds regression models, categorized by postgraduate year

**Sleep duration**

PGY-1

|  |  | **Unadjusted** | | **Adjusted*** | |
| --- | --- | --- | --- | --- | --- |
| **Duty hour category** | **N** | **Odds ratio**  **(95% CI)** | **P-value** | **Odds ratio**  **(95% CI)** | **P-value** |
| C1: < 50 hours | 369 | 1.46 (1.13–1.89) | < 0.001 | 1.59 (1.23–2.04) | < 0.001 |
| C2: ≥ 50 to < 60 hours | 633 | 1.29 (1.04–1.61) | 0.009 | 1.31 (1.06–1.61) | 0.01 |
| C3: ≥ 60 to < 70 hours | 635 | Reference | | | |
| C4: ≥ 70 to < 80 hours | 338 | 0.91 (0.71–1.18) | 0.26 | 0.90 (0.70–1.16) | 0.40 |
| C5: ≥ 80 to < 90 h | 395 | 0.97 (0.76–1.25) | 0.03 | 0.79 (0.62–1.01) | 0.06 |
| C6: ≥ 90 to < 100 hours | 112 | 0.75 (0.52–1.07) | < 0.001 | 0.48 (0.32–0.70) | < 0.001 |
| C7: ≥ 100 hours | 89 | 0.53 (0.40–0.71) | 0.006 | 0.62 (0.40–0.98) | 0.04 |

PGY-2

|  |  | **Unadjusted** | | **Adjusted*** | |
| --- | --- | --- | --- | --- | --- |
| **Duty hour category** | **N** | **Odds ratio**  **(95% CI)** | **P-value** | **Odds ratio**  **(95% CI)** | **P-value** |
| C1: < 50 hours | 385 | 1.46 (1.13–1.89) | 0.003 | 1.59 (1.22–2.06) | 0.001 |
| C2: ≥ 50 to < 60 hours | 613 | 1.29 (1.04–1.61) | 0.02 | 1.33 (1.07–1.66) | 0.01 |
| C3: ≥ 60 to < 70 hours | 542 | Reference | | | |
| C4: ≥ 70 to < 80 hours | 351 | 0.91 (0.71–1.18) | 0.48 | 0.94 (0.73–1.21) | 0.62 |
| C5: ≥ 80 to < 90 h | 375 | 0.97 (0.76–1.25) | 0.84 | 1.02 (0.79–1.31) | 0.87 |
| C6: ≥ 90 to < 100 hours | 137 | 0.75 (0.52–1.07) | 0.11 | 0.80 (0.56–1.14) | 0.22 |
| C7: ≥ 100 hours | 121 | 0.51 (0.35–0.75) | 0.001 | 0.56 (0.38–0.82) | 0.003 |

**Insomnia symptoms**

PGY-1

|  |  | **Unadjusted** | | **Adjusted*** | |
| --- | --- | --- | --- | --- | --- |
| **Duty hour category** | **N** | **Odds ratio**  **(95% CI)** | **P-value** | **Odds ratio**  **(95% CI)** | **P-value** |
| C1: < 50 hours | 369 | 0.62 (0.46–0.85) | 0.002 | 0.58 (0.41–0.82) | 0.002 |
| C2: ≥ 50 to < 60 hours | 633 | 0.88 (0.69–1.12) | 0.30 | 0.93 (0.72–1.22) | 0.62 |
| C3: ≥ 60 to < 70 hours | 635 | Reference | | | |
| C4: ≥ 70 to < 80 hours | 338 | 0.87 (0.65–1.16) | 0.33 | 0.80 (0.58–1.11) | 0.18 |
| C5: ≥ 80 to < 90 h | 395 | 0.89 (0.67–1.17) | 0.40 | 0.87 (0.64–1.17) | 0.36 |
| C6: ≥ 90 to < 100 hours | 112 | 1.64 (1.08–2.49) | 0.02 | 1.83 (1.18–2.84) | 0.007 |
| C7: ≥ 100 hours | 89 | 1.85 (1.17–2.92) | 0.009 | 1.56 (0.95–2.58) | 0.08 |

PGY-2

|  |  | **Unadjusted** | | **Adjusted*** | |
| --- | --- | --- | --- | --- | --- |
| **Duty hour category** | **N** | **Odds ratio**  **(95% CI)** | **P-value** | **Odds ratio**  **(95% CI)** | **P-value** |
| C1: < 50 hours | 385 | 0.87 (0.64–1.19) | 0.40 | 0.84 (0.60–1.18) | 0.32 |
| C2: ≥ 50 to < 60 hours | 613 | 0.86 (0.66–1.14) | 0.30 | 0.84 (0.63–1.13) | 0.25 |
| C3: ≥ 60 to < 70 hours | 542 | Reference | | | |
| C4: ≥ 70 to < 80 hours | 351 | 0.82 (0.59–1.13) | 0.22 | 0.79 (0.56–1.11) | 0.18 |
| C5: ≥ 80 to < 90 h | 375 | 0.99 (0.73–1.35) | 0.97 | 0.93 (0.67–1.28) | 0.66 |
| C6: ≥ 90 to < 100 hours | 137 | 1.24 (0.82–1.88) | 0.31 | 1.16 (0.74–1.80) | 0.53 |
| C7: ≥ 100 hours | 121 | 1.49 (0.97–2.30) | 0.07 | 1.16 (0.73–1.85) | 0.53 |

Note: The association between DHs and sleep durations, as well as between DHs and insomnia symptoms were analysed using proportional odds regression models with generalized estimating equations to account for hospital variability. The reference group for the analysis was duty hour category 3 (≥60 to 70 hours/week). Sleep duration was categorized into six categories per day: ≤4, 5, 6, 7, 8, and ≥9 hours. Insomnia symptoms was classified by scores on the Athens Insomnia Scale, with 0–5 points as “absence”, 6–9 points as “mild”, 10–15 points as “moderate”, and 16–24 points as “severe”.

*Both models are adjusted for age, sex, hospital type, emergency department duty, assigned inpatient, self-study time, and depressive symptoms.

Abbreviations: CI = confidence interval; PGY= postgraduate year.

**Supplementary Table 5.** Association between resident sleep and depressive symptoms analysed using modified Poisson regression models, categorized by postgraduate year

PGY-1

|  |  | **Unadjusted** | | **Adjusted*** | |
| --- | --- | --- | --- | --- | --- |
| **Duty hour category** | **N** | **Prevalence ratio**  **(95% CI)** | **P-value** | **Prevalence ratio**  **(95% CI)** | **P-value** |
| Sleep duration |  |  |  |  |  |
| < 4 hours | 37 | 2.53 (1.89–3.38) | < 0.001 | 2.19 (1.61–2.99) | < 0.001 |
| 5 hours | 387 | 1.57 (1.31–1.88) | < 0.001 | 1.50 (1.25–1.80) | < 0.001 |
| 6 hours | 1,271 | 1.09 (0.93–1.27) | 0.30 | 1.06 (0.91–1.24) | 0.45 |
| 7 hours | 751 | Reference | | | |
| 8 hours | 111 | 1.26 (0.93–1.72) | 0.14 | 1.23 (0.90–1.67) | 0.19 |
| > 9 hours | 13 | 1.31 (0.51–3.38) | 0.58 | 1.29 (0.48–3.42) | 0.61 |
| Insomnia symptoms† |  |  |  |  |  |
| Absence | 1,835 | Reference | | | |
| Mild | 555 | 2.94 (2.52–3.42) | < 0.001 | 2.88 (2.46–3.37) | < 0.001 |
| Moderate to severe | 181 | 5.05 (4.42–5.77) | < 0.001 | 4.92 (4.29–5.64) | < 0.001 |

PGY-2

|  |  | **Unadjusted** | | **Adjusted*** | |
| --- | --- | --- | --- | --- | --- |
| **Duty hour category** | **N** | **Prevalence ratio**  **(95% CI)** | **P-value** | **Prevalence ratio**  **(95% CI)** | **P-value** |
| Sleep duration |  |  |  |  |  |
| < 4 hours | 30 | 2.56 (1.72–3.83) | < 0.001 | 2.18 (1.47–3.22) | < 0.001 |
| 5 hours | 339 | 1.82 (1.48–2.23) | < 0.001 | 1.63 (1.32–2.01) | < 0.001 |
| 6 hours | 1,285 | 1.08 (0.89–1.32) | 0.43 | 1.04 (0.86–1.27) | 0.69 |
| 7 hours | 731 | Reference | | | |
| 8 hours | 121 | 1.00 (0.67–1.50) | 1.00 | 0.94 (0.62–1.41) | 0.75 |
| > 9 hours | 18 | 2.14 (1.22–3.76) | 0.008 | 1.90 (1.04–3.47) | 0.04 |
| Insomnia symptoms† |  |  |  |  |  |
| Absence | 1,902 | Reference | | | |
| Mild | 472 | 3.49 (2.98–4.08) | < 0.001 | 3.39 (2.89–3.97) | < 0.001 |
| Moderate to severe | 150 | 5.04 (4.28–5.94) | < 0.001 | 4.88 (4.11–5.79) | < 0.001 |

Note: The association between sleep-related variables (sleep duration and insomnia symptoms) and depressive symptoms were analysed using modified Poisson regression models with generalized estimating equations to account for hospital variability. Depressive symptoms were measured dichotomously with the Patient Health Questionnaire (PHQ)-2.

*Adjusted for age, sex, hospital type, weekly duty hour, emergency department duty, assigned inpatient, and self-study time.

†Insomnia symptoms was classified by scores on the Athens Insomnia Scale, with 0–5 points as “absence”, 6–9 points as “mild”, 10–15 points as “moderate”, and 16–24 points as “severe”. For the analysis, the “moderate” and “severe” categories were combined due to the small number of participants in the “severe” category (N = 45).

Abbreviations: CI = confidence interval; PGY= postgraduate year.

**Supplementary Table 4.** Association between resident sleep-related variables and medical errors analysed using proportional odds regression models, categorized by postgraduate year

**Minor error**

PGY-1

|  |  | **Unadjusted** | | **Adjusted*** | |
| --- | --- | --- | --- | --- | --- |
| **Duty hour category** | **N** | **Odds ratio**  **(95% CI)** | **P-value** | **Odds ratio**  **(95% CI)** | **P-value** |
| Sleep duration |  |  |  |  |  |
| < 4 hours | 37 | 0.90 (0.47–1.75) | 0.76 | 0.81 (0.41–1.63) | 0.56 |
| 5 hours | 387 | 0.92 (0.72–1.18) | 0.53 | 0.90 (0.70–1.17) | 0.44 |
| 6 hours | 1,271 | 0.99 (0.83–1.19) | 0.93 | 0.98 (0.81–1.18) | 0.83 |
| 7 hours | 752 | Reference | | | |
| 8 hours | 111 | 1.30 (0.87–1.94) | 0.20 | 1.31 (0.87–1.95) | 0.20 |
| > 9 hours | 13 | 0.39 (0.10–1.50) | 0.17 | 0.37 (0.09–1.14) | 0.15 |
| Insomnia symptoms† | |  |  |  |  |
| Absence | 1,835 | Reference | | | |
| Mild | 555 | 1.29 (1.07–1.57) | 0.009 | 1.27 (1.03–1.55) | 0.02 |
| Moderate to severe | 181 | 1.41 (1.03–1.92) | 0.03 | 1.32 (0.94–1.86) | 0.11 |

PGY-2

|  |  | **Unadjusted** | | **Adjusted*** | |
| --- | --- | --- | --- | --- | --- |
| **Duty hour category** | **N** | **Odds ratio**  **(95% CI)** | **P-value** | **Odds ratio**  **(95% CI)** | **P-value** |
| Sleep duration |  |  |  |  |  |
| < 4 hours | 30 | 1.33 (0.63–2.79) | 0.45 | 1.34 (0.64–2.79) | 0.44 |
| 5 hours | 339 | 0.93 (0.72–1.20) | 0.58 | 0.93 (0.71–1.21) | 0.57 |
| 6 hours | 1,285 | 0.90 (0.75–1.08) | 0.25 | 0.90 (0.75–1.08) | 0.25 |
| 7 hours | 731 | Reference | | | |
| 8 hours | 121 | 0.86 (0.58–1.27) | 0.45 | 0.96 (0.65–1.42) | 0.83 |
| > 9 hours | 18 | 1.06 (0.40–2.79) | 0.90 | 1.18 (0.45–3.14) | 0.74 |
| Insomnia symptoms† | |  |  |  |  |
| Absence | 1,902 | Reference | | | |
| Mild | 472 | 1.30 (1.07–1.59) | 0.01 | 1.18 (0.95–1.46) | 0.13 |
| Moderate to severe | 150 | 1.90 (1.37–2.63) | < 0.001 | 1.71 (1.21–2.42) | 0.002 |

**Severe error**

PGY-1

|  |  | **Unadjusted** | | **Adjusted*** | |
| --- | --- | --- | --- | --- | --- |
| **Duty hour category** | **N** | **Odds ratio**  **(95% CI)** | **P-value** | **Odds ratio**  **(95% CI)** | **P-value** |
| Sleep duration |  |  |  |  |  |
| < 4 hours | 37 | 1.78 (0.81–3.89) | 0.15 | 1.36 (0.59–3.11) | 0.47 |
| 5 hours | 387 | 1.00 (0.71–1.40) | 1.00 | 0.94 (0.66–1.33) | 0.71 |
| 6 hours | 1,271 | 1.03 (0.8–1.32) | 0.81 | 1.04 (0.81–1.34) | 0.75 |
| 7 hours | 752 | Reference | | | |
| 8 hours | 111 | 1.21 (0.72–2.04) | 0.71 | 1.18 (0.69–2.00) | 0.55 |
| > 9 hours | 13 | 2.74 (0.83–9.07) | 0.10 | 2.94 (0.88–9.79) | 0.08 |
| Insomnia symptoms† | |  |  |  |  |
| Absence | 1.835 | Reference | | | |
| Mild | 555 | 1.20 (0.93–1.55) | 0.16 | 1.16 (0.88–1.53) | 0.29 |
| Moderate to severe | 181 | 2.16 (1.51–3.10) | < 0.001 | 1.70 (1.13–2.57) | 0.01 |

PGY-2

|  |  | **Unadjusted** | | **Adjusted*** | |
| --- | --- | --- | --- | --- | --- |
| **Duty hour category** | **N** | **Odds ratio**  **(95% CI)** | **P-value** | **Odds ratio**  **(95% CI)** | **P-value** |
| Sleep duration |  |  |  |  |  |
| < 4 hours | 30 | 1.81 (0.78–4.24) | 0.17 | 1.78 (0.76–4.15) | 0.18 |
| 5 hours | 339 | 0.79 (0.56–1.10) | 0.17 | 0.74 (0.52–1.05) | 0.09 |
| 6 hours | 1,285 | 0.81 (0.65–1.02) | 0.08 | 0.80 (0.64–1.01) | 0.07 |
| 7 hours | 731 | Reference | | | |
| 8 hours | 121 | 1.02 (0.63–1.64) | 0.95 | 1.13 (0.70–1.83) | 0.62 |
| > 9 hours | 18 | 2.70 (0.98–7.41) | 0.05 | 3.20 (1.15–8.89) | 0.03 |
| Insomnia symptoms† | |  |  |  |  |
| Absence | 1.902 | Reference | | | |
| Mild | 472 | 1.37 (1.07–1.76) | 0.01 | 1.22 (0.93–1.60) | 0.15 |
| Moderate to severe | 150 | 2.78 (1.92–4.05) | < 0.001 | 2.29 (1.53–3.42) | < 0.001 |

Note: The association between sleep-related variables (sleep duration and insomnia symptoms) and medical errors were analysed using modified Poisson regression models with generalized estimating equations to account for hospital variability. The number of medical errors that resulted in minor or severe disability or disadvantage to patients experienced in the past year were reported based on the following numbers: 0, 1, 2, 3, 4, or 5 or more times.

*Adjusted for age, sex, hospital type, weekly duty hours, emergency department duty, assigned inpatient, self-study time, and depressive symptoms.

†Insomnia symptoms was classified by scores on the Athens Insomnia Scale, with 0–5 points as “absence”, 6–9 points as “mild”, 10–15 points as “moderate”, and 16–24 points as “severe”. For the analysis, the “moderate” and “severe” categories were combined due to the small number of participants in the “severe” category (N = 45).

Abbreviations: CI = confidence interval; PGY= postgraduate year.

**Supplementary Figure 1.** Results of structural equation modeling among the association between weekly duty hours, sleep-related variables, depressive symptoms, and minor medical error


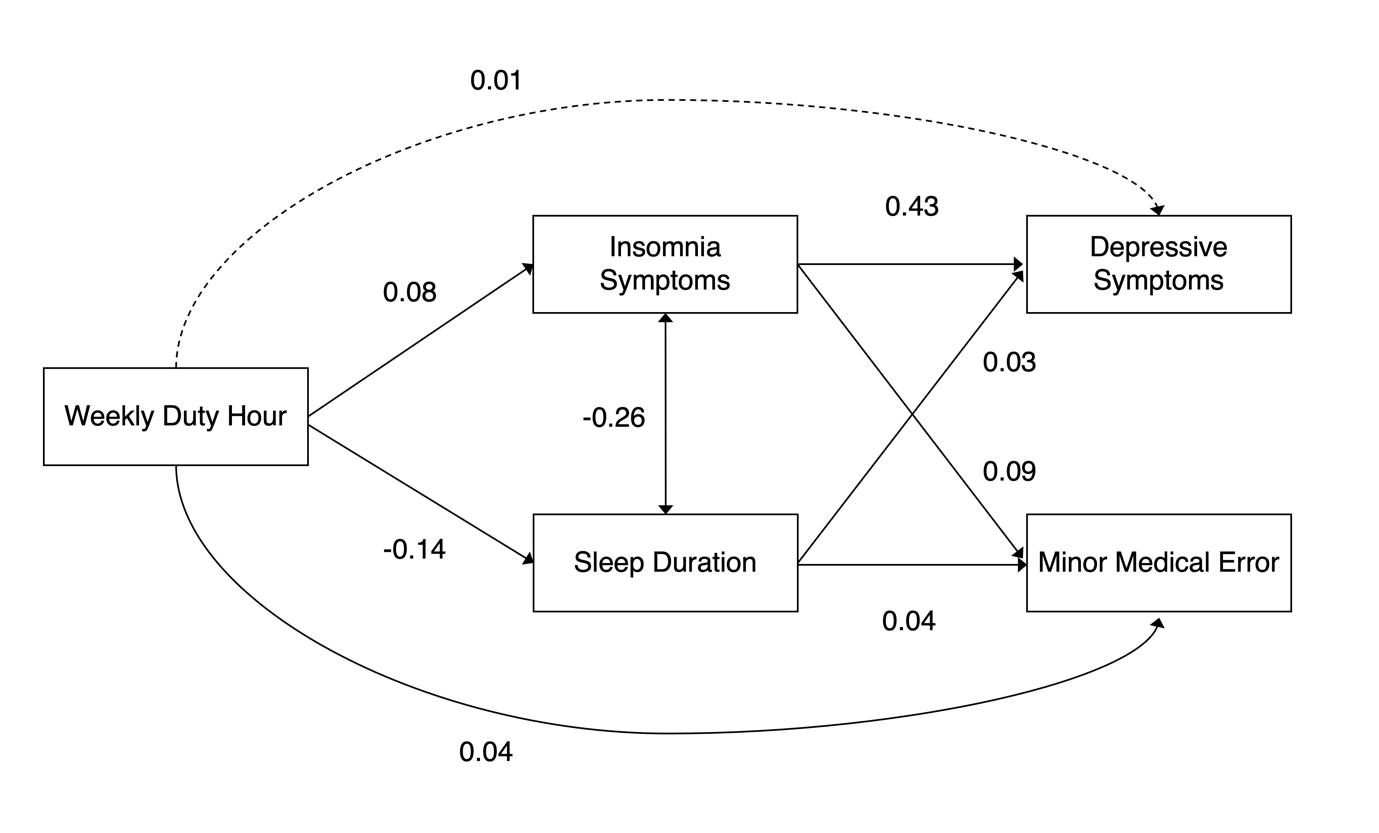


Note: Insomnia symptoms and sleep duration are considered as covariates. The values shown are standardized profit regression coefficients. Solid lines indicate statistical significance (p < 0.05), while dashed lines represent relationships that are not statistically significant. Our model was well fitted to our data (root mean square error of approximation [RMSEA]: 0.04; comparative fit index [CFI]: 0.995; Tucker-Lewis index [TLI]: 0.95).
